# Editorial: Molecular pathogenesis of enteroviruses: insights into viral-host interactions, pathogenic mechanisms, and microbiome dynamics

**DOI:** 10.3389/fmicb.2025.1608481

**Published:** 2025-05-08

**Authors:** Jade Lee Lee Teng, Shubhada Bopegamage, Man Lung Yeung

**Affiliations:** ^1^Faculty of Dentistry, The University of Hong Kong, Sai Ying Pun, Hong Kong SAR, China; ^2^Faculty of Medicine, Institute of Laboratory Medicine, Slovak Medical University, Bratislava, Slovakia; ^3^Department of Microbiology, School of Clinical Medicine, Li Ka Shing Faculty of Medicine, The University of Hong Kong, Pokfulam, Hong Kong SAR, China; ^4^Centre for Virology, Vaccinology and Therapeutics, Hong Kong Science and Technology Park, Pokfulam, Hong Kong SAR, China; ^5^Pandemic Research Alliance Unit, Li Ka Shing Faculty of Medicine, The University of Hong Kong, Pokfulam, Hong Kong SAR, China; ^6^State Key Laboratory of Emerging Infectious Diseases, Li Ka Shing Faculty of Medicine, The University of Hong Kong, Pokfulam, Hong Kong SAR, China; ^7^Carol Yu Centre for Infection, School of Clinical Medicine, Li Ka Shing Faculty of Medicine, The University of Hong Kong, Pokfulam, Hong Kong SAR, China

**Keywords:** enteroviruses, Picornaviridae, pathogenesis, viral-host interaction, microbiome

*Enterovirus*, a genus of small, non-enveloped RNA viruses within the Picornaviridae family, are among the most pervasive and clinically significant pathogens worldwide (Ooi et al., 2010; Wang et al., 2019). With over 100 serotypes identified—including poliovirus, coxsackieviruses, echoviruses, and enterovirus A71 (EV-A71)—these viruses exhibit remarkable genetic diversity and adaptability, enabling them to infect millions annually. Their clinical manifestations encompass a spectrum of acute neurological, respiratory, and systemic diseases, such as encephalitis, acute flaccid paralysis, meningitis, and hand, foot, and mouth disease (HFMD), as well as insidious links to chronic conditions like type 1 diabetes (T1D), chronic fatigue syndrome, and idiopathic dilated cardiomyopathy (Ho et al., 1999; Hsueh et al., 2000; Huang et al., 1999). The ability of enteroviruses to cause such a wide spectrum of diseases is attributed to their range of cell/organ tropism, capacity to evade the immune system (Pathinayake et al., 2015), persist in host tissues (Caine and Osorio, 2017), and exploit various cellular pathways for replication and dissemination (Good et al., 2019; Yeung et al., 2018).

Enteroviruses have been associated with severe outbreaks that pose significant public health challenges (Chang et al., 2016; Gilrane et al., 2020; Bubba et al., 2020). For instance, outbreaks of HFMD caused by EV-A71 have led to substantial morbidity and mortality globally (Puenpa et al., 2019), necessitating urgent public health responses and vaccine development efforts. The global distribution and high mutation rates of these viruses further complicate control measures, making it imperative to understand their molecular biology and pathogenesis comprehensively.

Globally, the public health systems grapple with emerging viral threats, understanding the molecular intricacies of enterovirus pathogenesis, host interactions, and microbiome dynamics is not just urgent—it is a cornerstone for developing targeted therapies and preventive strategies. This Research Topic, titled “*Molecular pathogenesis of enteroviruses: insights into viral-host interactions, pathogenic mechanisms, and microbiome dynamics*,” compiles original research articles that shed light on ongoing outbreaks in different geographical regions and identify factors that may contribute to the pathogenesis of enteroviruses.

## Outbreaks and epidemiological insights

Chen et al. study highlights the epidemiological trends of HFMD and herpangina (HA) in Zhejiang Province between 2021 and 2023. They documented 47 outbreaks, primarily in childcare settings, with a notable shift in HFMD strains from coxsackievirus A16 (CVA16) to coxsackievirus A6 (CVA6) and an association of coxsackievirus A4 (CVA4) with HA. The study emphasizes the need for timely intervention strategies, including early case isolation, to control these outbreaks and mitigate their impact on educational settings.

Similarly, Zhou et al. reported on HFMD reinfection patterns in Jiulongpo District, Chongqing, from 2009 to 2023. They identified a reinfection rate of 5.48%, with most cases occurring within 2 years. The study also found higher reinfection rates among males, kindergarten children, older children, and those in rural areas, suggesting the need for targeted interventions to improve sanitation and disease awareness.

Expanding beyond China, Xie et al. examined the epidemiology and molecular characteristics of enterovirus infections in children hospitalized with acute gastroenteritis (AGE) in Chiang Mai, Thailand, from 2019 to 2022. They found a significant decrease in infection rates and genotype diversity post-COVID-19, with coxsackievirus A2 (CVA2) emerging as a predominant genotype. This study provides valuable insights into the molecular epidemiology of enteroviruses in AGE patients.

## Molecular mechanisms and host interactions

Coxsackievirus infections have been associated to T1D, which involve triggering mechanisms leading to autoimmune destruction process of insulin-producing beta cells. In Bonfim et al. study, the authors explored the role of N6-methyladenosine (m6A) RNA modification in coxsackievirus B1 (CVB1) replication. They found that downregulating m6A writers increased CVB1 replication, while downregulating m6A erasers decreased it. This highlights the critical role of m6A in CVB1 replication and suggests that targeting the m6A machinery could be a potential therapeutic strategy for controlling coxsackievirus B (CVB) infections and subsequently T1D.

## Extracellular vesicles and viral spread

Accumulated evidence supports that the extracellular vesicles play a crucial role in intercellular communication, facilitating the transfer of bioactive molecules that influence various physiological and pathological processes. In the context of viral infections, such as porcine epidemic diarrhea virus (PEDV) and EV-A71, understanding the role of extracellular vesicles becomes paramount. Wu et al. demonstrated that PEDV infection can lead to abnormal regulation of microRNAs within exosomes isolated from infected porcine small intestine tissue, shedding light on the intricate interplay between the virus and the host and uncovering potential therapeutic targets. Moreover, viruses like EV-A71 exploit extracellular vesicles for viral dissemination. Mao et al. investigated the role of lipid signaling in extracellular vesicles from EV-A71-infected cells. They found a significant increase in lipid content in EVs post-infection, suggesting that altered lipid profiles in EVs from EV-A71-infected cells may impact their function in recipient cells.

## Conclusion

In summary, this Research Topic highlights the significance of enteroviruses. The scientific evidence presented in this Research Topic has identified key factors that may contribute to the pathogenesis of enterovirus-related diseases, further emphasizing the importance of these viruses in light of the ongoing outbreak. This new knowledge can enhance our understanding and development of effective disease management strategies to combat these critical viral infections.
